# Ocular perfusion pressure and color Doppler imaging of the external ophthalmic artery of rabbits treated with sildenafil citrate

**DOI:** 10.1186/s12917-016-0778-5

**Published:** 2016-07-22

**Authors:** Ana Paula Araujo Costa, Aline Maria Vasconcelos Lima, Luiz Henrique da Silva, Rosângela de Oliveira Alves Carvalho, Andréia Vitor Couto do Amaral, Naida Cristina Borges

**Affiliations:** Department of Veterinary Medicine, School of Veterinary Medicine and Animal Science, Veterinary Hospital, Federal University of Goiás, Campus Samambaia, Caixa Postal 131, Goiânia, Goiás State CEP 74001-970 Brazil; Department of Veterinary Medicine, School of Veterinary Medicine and Animal Science, Federal University of Goiás, Jataí, GO Brazil

**Keywords:** Phosphodiesterase type 5 inhibitor, Color Doppler imaging, Intraocular pressure, Mean arterial pressure, Rabbit, Eye circulation

## Abstract

**Background:**

It has been proposed that sildenafil citrate can increase ocular blood flow, and that this property can be used to treat ocular disorders that involve reflex vasoconstriction. This study therefore proposes to ascertain the vasodilator effect of the drug on retrobulbar circulation in healthy rabbits. For this matter rabbits treated with sildenafil citrate or saline solution had their intraocular pressure (IOP), mean arterial pressure (MAP), ocular perfusion pressure (OPP) and color Doppler imaging of the external ophthalmic artery measured prior to treatment and on days one (moment M1), seven (when M2), fourteen (moment M3), twenty-one (moment M4), and thirty (moment M5) of treatment.

**Results:**

The MAP and OPP values of treated group were lower than those of control group at all times, and the mean values differed statistically at moments M1 (S = 71.52 mmHg, C = 84.76 mmHg, *p* = 0.0356) and M5 (S = 71.38 mmHg, C = 85.52 mmHg, *p* = 0.0252). The IOP and color Doppler values of the external ophthalmic artery did not differ between tested groups.

**Conclusions:**

The dose of 10 mg of sildenafil citrate administered to healthy rabbits causes systemic vasodilation and consequently lower values of MAP and OPP. However, it does not induce changes in IOP and retrobulbar hemodynamics identifiable by color Doppler assessment of the external ophthalmic artery.

## Background

Sildenafil citrate was the first phosphodiesterase type 5 (PDE-5) inhibitor to be approved for treatment of erectile dysfunction, and has become one of the world’s most widely prescribed drugs. This drug inhibits PDE5, enzyme that hydrolyzes cyclic guanosine monophosphate (cGMP), regulating the circulating levels of cGMP, which in turn causes the corpus cavernosum muscles to relax and local blood flow to increase [1, 2].

PDE5, however, is present not only in the corpus cavernosum penis but also in the cells of the smooth muscle of peripheral arteries and veins, and in the pulmonary and coronary circulation, platelets and endothelial cells of blood vessels [3]. Therefore, PDE5 inhibitor drugs can cause systemic hemodynamic changes [2].

Although the drug is widely used for treatment of erectile dysfunction, some users exhibited ocular side effects such as blurred vision and increased sensitivity to light. These symptoms are associated with cross-inhibition of the drug with phosphodiesterase type 6 that is present in the retina, which participates in the regulation of the phototransduction mechanism. However, no consensus has yet been reached about the effects of sildenafil citrate on retinal and retrobulbar blood flow upon inhibiting the PDE5 contained in the walls of the vessels in these regions [2].

According to Silva et al. [4], identifying and quantifying the vasodilator activity in retrobulbar circulation resulting from the action of sildenafil citrate can provide important clinical information, especially for patients suffering from ocular reflex vasoconstriction conditions, such as retinal hypertension observed in patients with kidney failure. According to this author, chronic hypertension leads to continuous compensatory vasoconstriction of retinal arterioles, inducing ischemia and retinal degeneration, which impairs the functioning of this tissue.

Considering the potential of sildenafil citrate for treatment of ocular conditions with reflex vasoconstriction, the purpose of this study was to determine whether the drug has a vasodilator effect on retrobulbar circulation in healthy rabbits, based on an assessment of ocular perfusion pressure and on color Doppler imaging of the external ophthalmic artery.

## Methods

### Animals: care, husbandry, and general experimental procedure

The experiment was based on the recommendations of the Association for Research in Vision and Ophthalmology (ARVO) and Animal Research: Reporting of in Vivo Experiment (ARRIVE) guidelines. The study was also submitted under Proposal No. 027/12, and was approved by the Ethics Committee on Animal Use of the Federal University of Goiás (UFG).

The study involved 14 healthy adult male New Zealand white rabbits weighing on average 2.5 kg. The sample size was based on the number required to obtain reliable statistical results. The animals were acquired from a local supplier (MH Cunicultura Coelho Forte), located at Rodovia Bela Vista-Hidrolândia Km 9, Bela Vista de Goiás, Goiás – Brazil. The animals’ eyes and overall health were considered normal after a clinical examination done by an experienced veterinarian, as recommended by TALIERI et al. [5]. The animals were kept in a bioterium, under veterinary supervision, and transported to the laboratory when needed for the experiments. In the bioterium, the rabbits were housed in individual cages with free access to food and water, and the local temperature was kept at 24 ± 1 °C.

The rabbits were divided into two groups of seven animals each (*n* = 7), a control group (C) and a treatment group (S). Group (C) was given 1.5 mL of saline orally, while group (S) was given 10 mg of sildenafil citrate (Viagra®, Pfizer, Guarulhos, SP) orally. Both groups were treated at 24-h intervals for 30 consecutive days.

The experimental protocol followed the sequence: (1) oral administration of the vasodilator sildenafil, (2) measurement of intraocular pressure (IOP), (3) measurement of mean arterial pressure (MAP), and (4) color Doppler imaging of the external ophthalmic artery. Because sildenafil citrate was administered orally, a 45 to 60 min wait time was allowed between ingestion of the drug and the subsequent steps of the protocol.

The evaluations were performed weekly, as follows: Day one - moment 1 (M1), Day seven - moment 2 (M2), Day fourteen - moment 3 (M3), Day twenty-one - moment 4 (M4), and day thirty - moment 5 (M5).

To measure the IOP and MAP and for the Doppler study, the unsedated rabbits were wrapped in a towel, leaving only the head exposed for the investigator to take the measurements. Care was taken not to apply excessive force in restraining the animals during these evaluations.

All the evaluations were performed in triplicate by the same investigator, in a blind study. To reduce the period of restraint, only the right eye of each animal was examined, thus avoiding the effects of stress on the retrobulbar circulation and optimizing the action time of the drug.

### Measurement of IOP

The intraocular pressure of rabbits was measured with a Tono-Pen AVIA VET® applanation tonometer (Reichert®, New York, USA). The procedure consisted in gently lifting the eyelid, applying a drop of 0.5 % proparacaine (Anestalcon®, Alcon, São Paulo, SP) on the eye, and taking a reading with the tonometer five minutes later.

### Measurement of MAP

After the trichotomy and antisepsis of the dorsal surface of the rabbits’ ears, the central artery was cannulated with a 24G catheter (BD Angiocath^TM^, Becton Dickinson Indústrias Cirúrgicas Ltda, Juiz de Fora, MG, Brazil). The catheter was then plugged to a semi-rigid silicone tubing system. In this system, two silicone tubes were connected to a three-way stopcock. The free end of one the tube was attached a catheter and the other end to a BD sphygmomanometer (Becton, Dickinson and Company, Franklin Lakes, NJ, USA). The system was then filled with 1 mL/1000 mL of 0.9 % heparinized saline (Heparin, Cristália Produtos Químicos Farmaceuticos Ltda, Itapira, SP, Brazil). The air/liquid interface was positioned at the height of the right atrium and eye, and the mean arterial pressure was read with the sphygmomanometer [6].

### Calculation of OPP

The ocular perfusion pressure (OPP) was determined by subtracting the mean arterial pressure (MAP) from the intraocular pressure (IOP), as described by KIEL & HEUVEN [6].

### Color Doppler imaging

The color Doppler evaluation of the external ophthalmic artery was performed using a MyLab™ 30 VET ultrasound system (The Esaote Group, Genoa, Italy) coupled to a 13–18 MHz linear transducer.

Before the examination, the cornea was anesthetized by applying one drop of 0.5 % proparacaine hydrochloride topical ophthalmic anesthetic (Anestalcon®, Alcon, São Paulo, SP, Brazil). A layer of sterile aqueous gel was then applied to the corneal surface, and the transducer was gently placed in a longitudinal position, with the position indicator facing the upper eyelid.

The sagittal image of the eyeball and optic nerve were recorded in two-dimensional mode. The external ophthalmic artery was identified near the entrance of the optic nerve by color Doppler imaging. The cursor of the pulsed wave Doppler was then immediately positioned over the ophthalmic artery, within the vessel lumen, using the uniform insonation method, to record its blood flow curve, after which the peak systolic velocity (PSV) and end diastolic velocity (EDV) were evaluated based on this curve. The angle was not corrected and measurements greater than 60° were not included. The ophthalmic artery resistance index (RI) was calculated, automatically by the software Mylab desk, witch in based on the Pourcelot equation (RI = PSV − EVS/ PSV), and the curves were analyzed by the same operator (APAC), whom marked the PSV and the EDV of three consecutive curves [7].

### Statistical analysis

A randomized 2 x 5 split-plot experimental design was used, with the treatments corresponding to the plots (with and without sildenafil citrate), the evaluation periods to the subplots (M1 - M2 - M3 - M4 - M5), and each animal to an experimental unit.

Using R® statistical software, the data were tested for normality by the Shapiro-Wilk test and subjected to an analysis of variance. The means were compared by the Tukey test, adopting a 5 % level of significance.

## Results

### Ocular perfusion pressure

The mean baseline IOP, MAP and OPP values were 11.85 mmHg, 80.71 mmHg and 68.85 mmHg, respectively, in both groups.

In experimental conditions, the MAP and OPP values of group (S) were lower and statistically different from those of group (C) at moments M1 (S = 71.52 mmHg, C = 84.76 mmHg, *p* = 0.0356) and M5 (S = 71.38 mmHg, C = 85.52 mmHg, *p* = 0.0252). However, the IOP values did not differ statistically during the experimental period (Table 1).

**Table 1:** Means and standard errors of measurement of mean arterial pressure (MAP), intraocular pressure (IOP) and ocular perfusion pressure (OPP) of rabbits treated with 10 mg of sildenafil citrate (sildenafil) and not treated (control) for 30 days

Moments	Treatment		*P* values		
	Sildenafil	Control	P^*^ (T)	P^**^ (M)	P^***^ (I)
MAP					
M1	71.52 (4.32)^Ab^	84.76 (4,32) ^Aa^	0.0254	0.9604	0.4547
M2	74.95 (4.32) ^Aa^	84.85 (4.32) ^Aa^			
M3	78.71 (4.32) ^Aa^	80.09 (4.32) ^Aa^			
M4	75.14 (4.32) ^Aa^	78.95 (4.32) ^Aa^			
M5	71.38 (4.32) ^Ab^	85.52 (4.32) ^Aa^			
IOP					
M1	11.52 (0.79) ^Aa^	10.66 (0.79) ^Aa^	0.0834	0.4102	0.9989
M2	13.04 (0.79) ^Aa^	12.04 (0.79) ^Aa^			
M3	12.24 (0.79) ^Aa^	11.14 (0.79) ^Aa^			
M4	12.57 (0.79) ^Aa^	11.23 (0.79) ^Aa^			
M5	12.43 (0.79) ^Aa^	11.38 (0.79) ^Aa^			
OPP					
M1	59.99 (4.52) ^Ab^	74.09 (4.52) ^Aa^	0,0175	0,9744	0,5525
M2	61.90 (4.52) ^Aa^	72.81 (4.52) ^Aa^			
M3	66.47 (4.52) ^Aa^	68.95 (4.52) ^Aa^			
M4	62.57 (4.52) ^Aa^	67.71 (4.52) ^Aa^			
M5	58.95 (4.52) ^Ab^	74.14 (4.52) ^Aa^			

M = moment. M1 = day one, M2 = day seven, M3 = day fourteen, M4 = day twenty-one, M5 = day thirty

Treatment P * (T), P ** Moment (M) P *** Interaction (I)

Tukey test at a 5 % level of significance. The same letters correspond to the same means, with upper case letters representing moments within the same treatment and lower case letters to moments of different treatments. Values in brackets show standard errors of the means

### Color Doppler imaging

**Fig. 1:**
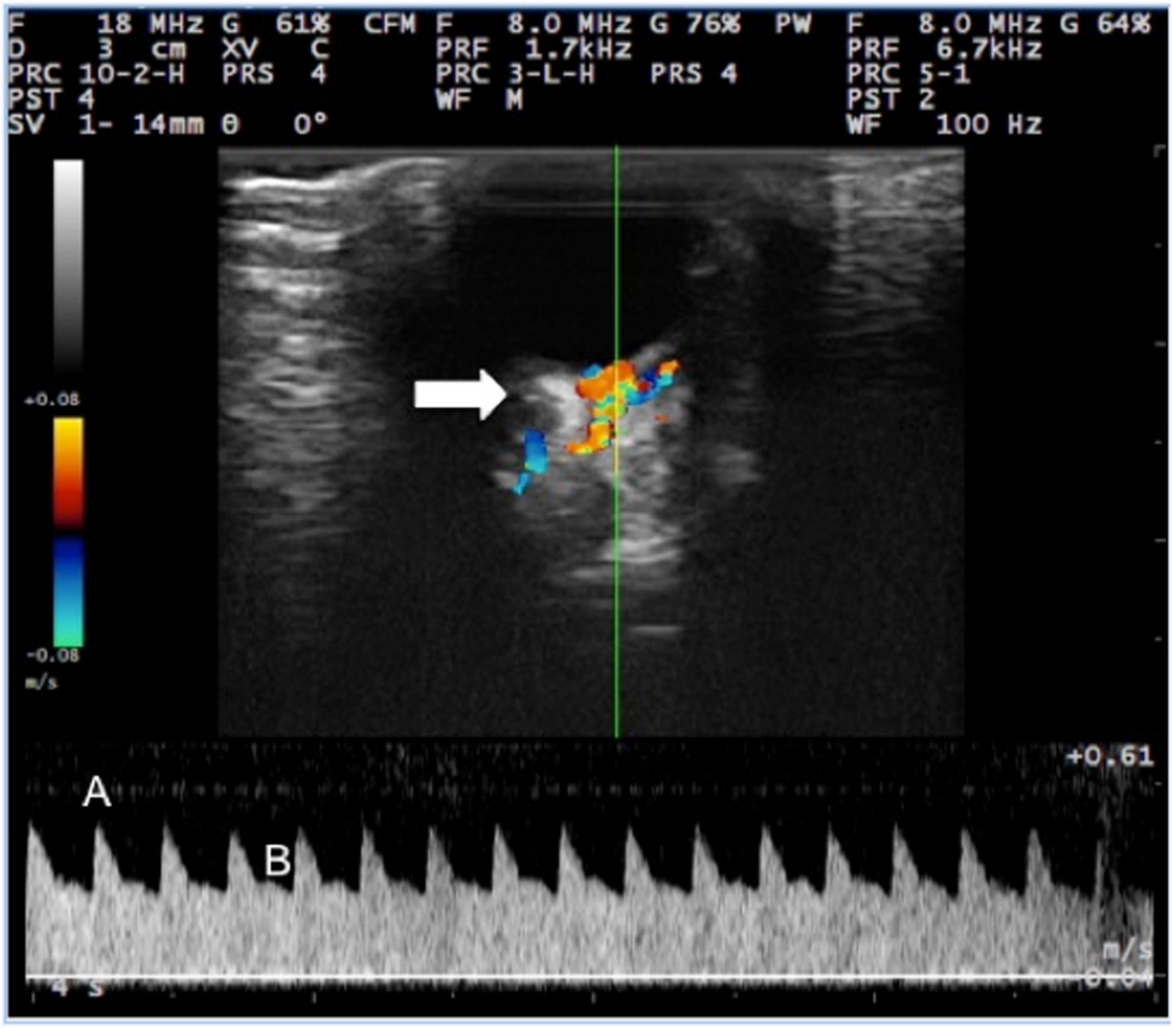
Color Doppler image and pulsed wave flow of the external ophthalmic artery of an adult male New Zealand white rabbit treated with 10 mg of sildenafil citrate (day 15). Blood flow in the external ophthalmic artery towards the transducer shown in red and flow in the opposite direction in blue. The external ophthalmic artery exhibited a laminar flow pattern with intermediate resistivity, dichroism, and two peak systolic velocities (**a** and **b**). Optic nerve (*white arrow*)

Prior to treatment, the mean baseline values observed in color Doppler study of the external ophthalmic artery of the right eye of rabbits in both groups were 22.99 cm/s at PSV and 12.29 cm/s at EDV and the resistance index was 0.53. The external ophthalmic artery showed a red flow in the color Doppler and exhibited a laminar flow pattern with intermediate resistivity, dichroism, and the presence of two peak systolic velocities (Fig. 1). Under experimental conditions, however, the color Doppler values of tested groups showed no statistical difference (Table 2).

**Table 2:** Means and standard errors of measured peak systolic velocity (PSV), end diastolic velocity (EDV) and resistance index (RI) of the external ophthalmic artery of rabbits treated with 10 mg of sildenafil citrate (sildenafil) and untreated rabbits (control) in 30 days

Moments	Treatment		P* Values		
	Sildenafil	Control	P^*^ (T)	P^**^ (M)	P^***^ (I)
PSV					
M1	28.92 (1,79)^Aa^	28.62 (2,06)^Aa^			
M2	32.06 (1,79)^Aa^	26.55 (1,79)^Aa^			
M3	29.49 (1,79)^Aa^	26.30 (2,05)^Aa^	0.265	0.126	0.364
M4	29.26 (1,90)^Aa^	28.85 (1,79)^Aa^			
M5	26.19 (1,79)^Aa^	25.30 (1,79)^Aa^			
EDV					
M1	14.64 (1.25)^Aa^	16.54 (1.44)^Aa^			
M2	17.37 (1.25)^Aa^	14.05 (1.25)^Aa^			
M3	15.19 (1.25)^Aa^	14.47 (1.43)^Aa^	0.691	0.177	0.18
M4	15.94 (1.33)^Aa^	15.53 (1.25)^Aa^			
M5	13.53 (1.25)^Aa^	13.56 (1.25)^Aa^			
RI					
M1	0.48 (0.01)^Aa^	0.47 (0.02)^Aa^			
M2	0.46 (0.01)^Aa^	0.46 (0.01)^Aa^			
M3	0.49 (0.01)^Aa^	0.44 (0.02)^Aa^	0.379	0.811	0.54
M4	0.45 (0.02)^Aa^	0.46 (0.01)^Aa^			
M5	0.48 (0.01)^Aa^	0.46 (0.01)^Aa^			

M = moment. M1 = day one, M2 = day seven, M3 = day fourteen, M4 = day twenty-one, M5 = day thirty

Treatment P * (T), P ** Moment (M) P *** Interaction (I)

Tukey test at a 5 % level of significance. The same letters correspond to the same means, with upper case letters representing moments within the same treatment and lower case ones to moments of different treatments. Values in brackets show standard errors of the means

## Discussion

In this study, it was found that sildenafil citrate reduced mean arterial pressure in rabbits. In humans, this drug has also been found to cause a significant reduction in systolic and diastolic blood pressure one hour after ingestion [8, 9]. However, other studies found no changes in blood pressure in humans after the administration of this drug [10–12]. Despite the significant decrease in MAP at moments M1 and M5, the treated animals showed no hypotension, given that the normal blood pressure of this species ranges from 70 mmHg to 170 mmHg [13].

As for intraocular pressure, no statistical differences were found between the groups. Like this study, other studies on rabbits have shown no significant increases in IOP with the use of sildenafil citrate [14–16]. However, sheep treated with the minimum (50 mg) and maximum (100 mg) doses recommended for humans showed an elevation of IOP [17]. Another study also reported increased IOP in humans one hour after ingestion of the drug, with a return to baseline values in the second hour [9]. Other studies involving healthy humans with open angle glaucoma did not find that the drug affected the IOP values [8, 10, 18]. The MAP is known to have a minimal effect on IOP, since the production of aqueous humor depends on three phenomena, namely, ultrafiltration, active secretion and diffusion [19], and therefore minor decreases in MAP will not change the IOP, as noted in the study.

In this study, the ocular perfusion pressure of rabbits was calculated based on measured MAP and IOP values. The perfusion pressure of an organ can be defined as the difference between its arterial and venous blood pressure [20]. In rabbits, the mean ophthalmic arterial pressure is similar to the MAP, while the mean ophthalmic venous pressure is similar to the IOP [6]. The ocular perfusion pressure (OPP) is therefore determined from the difference between MAP and IOP [20]. Thus, the increase in IOP or the decrease in MAP may lower the perfusion pressure of the tissue of the eye [21]. The animals used in this study presented significant decreases in MAP which resulted in decrease in OPP, with groups (C) and (S) showing a statistical difference at moments M1 and M5. In human patients with age-related macular degeneration, sildenafil citrate also reduced the OPP by reducing the MAP, associated with the maintenance of the IOP within normal values [8].

The ophthalmic artery resistance index of rabbits did not change in response to sildenafil citrate. Moreover, it has been shown that the drug does not alter the resistance of the ophthalmic artery in humans [22]. Another study, however, reported increased resistance of the ophthalmic artery one hour after the administration of 100 mg of sildenafil citrate in men with erectile dysfunction [11]. Bioequivalence studies of sildenafil citrate done in rabbits, humans and rats showed that the distribution volume in rabbits is similar to humans, however the greater hepatic metabolism in rabbits can diminish its bioavailability. Also, the greater renal excretion in rabbits can diminish the half-life of sildenafil citrate in this species [23]. Knowing that, the authors of this paper choose to use a greater dose than the human dose (50–100 mg/human or 0,7–1,4 mg/Kg in a 70 Kg human) and a dose the have shown increase retinal circulation in rabbits (3,5 mg/Kg or 10 mg/rabbit) in a previous study preformed by the co-autor AVCA in 2014 [16].

Although the decrease in MAP was expected to lower the external ophthalmic artery resistance index, this was not observed in this study. It is believed that this result may be due to compensatory mechanisms of self-regulation that act on the external ophthalmic artery to maintain the ocular infusion [11], or that sildenafil citrate does not act on the muscles of the external ophthalmic artery. Tissues that are sensitive to the action of sildenafil citrate are innervated by nitric oxide-producing neurons. Nitric oxide is a potent vasodilator that, when released, stimulates endogenous receptors to release cGMP, a second messenger that triggers a cascade of smooth muscle relaxation of the vessels. The PDE5 enzyme degrades the excess of cGMP. Sildenafil inhibits PDE5, increasing the cGMP levels and causing vasodilation [24]. PDE5 expression has been reported in human retinal and choroidal vasculature [12]. However, no studies were found in the literature about the presence of PDE5 in the ophthalmic artery of humans or in the external ophthalmic artery of rabbits; hence, it is not possible to state whether or not this vessel is sensitive to the action of sildenafil citrate.

## Conclusions

In summary, based on the data obtained in this study, it can be stated that a dose of 10 mg of sildenafil citrate administered to healthy rabbits causes systemic vasodilation, lowering their mean arterial pressure and ocular perfusion pressure. However, the drug does not induce changes in the retrobulbar hemodynamics detectable by color Doppler imaging of the external ophthalmic artery, nor does it alter the IOP.

## Abbreviations

ARVO, Association for Research in Vision and Ophthalmology; cGMP, cyclic guanosine monophosphate; EDV, end diastolic velocity; IOP, intraocular pressure; MAP, mean arterial pressure; OPP, ocular perfusion pressure; PDE-5, phosphodiesterase type 5; PSV, peak systolic velocity; RI, resistance index
